# Inducing salt stress tolerance in bitter gourd (*Momordica chanrantia*) through seed treatment with chitosan

**DOI:** 10.3389/fpls.2024.1525561

**Published:** 2025-01-31

**Authors:** Ahsan Ali, Fiaz Hussain Ferdosi, Mubeen Sarwar, Sumreen Anjum, Zain Mushtaq, Mehwish Liaquat, Muhammad Taqqi Abbas, Moazzam Anees, Muhammad Rizwan Tariq, M. Irfan Ashraf, Abdulrahman Alasmari, Md Sabir Ahmed Mondol, Gholamreza Abdi

**Affiliations:** ^1^ Department of Horticulture, University of the Punjab, Lahore, Pakistan; ^2^ Institute of Botany, University of the Punjab, Lahore, Pakistan; ^3^ Department of Soil Science, University of the Punjab, Lahore, Pakistan; ^4^ Department of Horticulture, PMAS Arid Agriculture University, Rawalpindi, Pakistan; ^5^ Department of Plant Pathology, University of the Punjab, Lahore, Pakistan; ^6^ Department of Food Science, University of the Punjab, Lahore, Pakistan; ^7^ Institute of Horticultural Sciences, University of Agriculture, Faisalabad, Faisalabad, Pakistan; ^8^ Biology Department, Faculty of Science, University of Tabuk, Tabuk, Saudi Arabia; ^9^ Department of Agricultural Biochemistry, Bidhan Chandra Krishi Viswavidyalaya, Mohanpur, West Bengal, India; ^10^ Department of Biotechnology, Persian Gulf Research Institute, Persian Gulf University, Bushehr, Iran

**Keywords:** salt stress, chitosan, yield attributes, bitter gourd, seed treatment

## Abstract

**Background:**

Bitter gourd requires well-drained sandy to sandy loam soils for optimum growth, development, and germination, while its growth is retarded in extreme saline conditions. It is very sensitive to salinity stress, which imposes devastating limits on its productivity. Thus, the impact of soil salinization on the economics of bitter gourd yield deserves scientific inquiry.

**Methods:**

The present study was designed to evaluate the various morphological attributes (mean germination time, germination index, final emergence percentage, measurements of root length, measurement of shoot length, measurement of plant dry biomass, and measurement of plant fresh biomass), physiological attributes (leaf chlorophyll content and electrolyte leakage), biochemical attributes (proline contents, antioxidant enzymes, superoxide dismutase, catalase Q9 , and peroxidase), leaf water relations (leaf osmotic potential, leaf water potential, leaf turgor potential, and leaf relative water content), and ion concentrations (Na+, K+, Ca +, and Cl-) that can be used for the evaluation of salt stress tolerance potential in bitter gourd. The research was conducted in the field area of the Faculty of Agricultural Sciences, University of the Punjab, Lahore.

**Results:**

In this experiment, bitter gourd seeds were sowed either without treatment or with hydropriming, 0.01%, 0.02%, 0.03%, 0.04%, and 0.05% chitosan, respectively, under 50mM soil salinity under the climatic conditions of Lahore. This research was designed to find the role of chitosan in inducing salt stress tolerance in bitter gourd plants and also find the best chitosan dose that is useful for higher salinity conditions. Different attributes of bitter gourd were recorded. Results revealed that chitosan application at 0.04% is best for enhancing the salt stress tolerance potential of bitter gourd. Different morphological attributes, physiological attributes, water relation attributes, and biochemical parameters were also recorded. It was observed that pre-sowing treatments with an optimized dose of 0.04% chitosan exhibited significant effects on all the bitter gourd plants and improved the germination rate by improving the salt stress tolerance potential of plants under high salinity.

**Conclusion:**

It can be concluded from the present research that the optimized dose of 0.04% chitosan has also proved effective in the enzymatic activity of bitter gourd by enhancing the salt stress potential under increasing salt stress.

## 1 Introduction

Bitter gourd (*Momordica charantia*) is a highly nutritious and therapeutic vegetable that is often utilized when it is still young. It is a rich source of vitamins (C and A), minerals, and dietary fibers (Baig et al., 2020). Additionally, it purifies the blood, which is highly advantageous for those with diabetes (Gopalan et al., 1976; Adhikari et al., 2021). A cytostatic treatment for several cancers, bitter gourd also possesses anti-carcinogenic qualities (Yibchok-Anun et al., 2006). It is traditionally used to treat a number of microbiological diseases, hyperlipidemia, menstrual issues, and digestive ailments (Gopalan et al., 1976). All vegetables contain sources of minerals and phytochemicals that are crucial for a variety of bodily metabolic processes (Noreen and Ashraf, 2009). However, the deterioration of cultivated fields due to an increase in salt buildup poses a threat to the production of vegetables, especially in irrigated regions, which provide 40% of the world’s food (Unver, 2010).

Environmental degradation, rising soil salinization, and a lack of water resources pose a serious danger to food security and agricultural sustainability in the twenty-first century (Shahbaz and Ashraf, 2013). The primary barriers to food security are abiotic stressors. Abiotic challenges such as salt, drought, high temperature stress, and cold injury have negatively affected the development and production of food plants, making them less likely to survive (Muhammad Jamil et al., 2006). Salt stress is one of the main abiotic stresses that is impeding agricultural yields globally (Muhammad Jamil et al., 2006). According to Fahmi et al. (2011), approximately 900 million hectares of global lands are disturbed by sodic and saline conditions, which affects approximately 7% of the world’s total area (Munns and Tester, 2008). Salinity has an impact on 40% of agricultural lands and 50% of irrigated regions (Roy and Chakraborty, 2014).

High concentrations of salts in the rhizosphere induce impaired seed emergence and crop establishment owing to osmotic pressure, making germination the most vulnerable period in the life of angiosperms (Rashid et al., 2006). Seed germination under saline and alkaline soils is crucial because of crust formation and hard setting (Dutta, 2018), with poor plant populations and weak seedlings that are susceptible to diseases and pests as the major consequences. Finally, it caused low economic yields (Basra et al., 2005). Salt stress mainly showed an impact on water uptake, reduced the cell turgor, and depressed the leaf/root elongation rate (Fricke et al., 2006). Furthermore, higher amounts of Na^+^ and Cl^-^ ions intracellularly can hamper the metabolism of cell division and cell exception, delaying emergence and leading to seed death. Thus, in this situation, seed treatment improves the germination, germination percentage, activates protein synthesis (Farooq et al., 2004), and starts the synthesis of enzymes that participate in cell metabolism (Varier et al., 2010).

Bitter gourd seeds have a strong germination capacity, however, in field conditions, their field emergence is usually problematic because of the seeds’ thick seed coat, which progressively results in poor germination (Asna et al., 2020). To overcome the problem of seed germination and uniform emergence, there is several tools have been practiced but seed priming is one of the best techniques. Seed treatment is a simple and economical technique and a viable strategy for improving seed emergence in crops (Farooq et al., 2019). It is termed a physiological approach that involves hydration and drying of seeds to improve the pre-germinative metabolic process without radicle protrusion in water or a solution of other priming agents (Sher et al., 2019). Many horticultural crops have presented a beneficial response to this seed treatment and this ultimately improves the crop productivity (Basra et al., 2005). Early emergence and uniform germination through the breakdown of photo and thermo dormancy with an extended germination temperature range and maximum nutrient uptake have been described as the benefits of seed priming (Ahmad et al., 2015). It is reported that early growth stages are as sensitive as the later stages of plant growth (Hussain et al., 2011). The salt stress tolerance mechanism in seeds is very intricate, especially when it is compared with recent available information about the physiological and biochemical basis of salt tolerance in plants (Kanai et al., 2007).

The deadly effect of salt stress on productivity has been mitigated by a number of methods (Ashraf et al., 2008). Reclamation of soil with the application of gypsum is the most prominent throughout the world, including Pakistan (Cha-Um et al., 2011), but it is a very costly approach. In order to increase plant growth under salty soils, the excessive salts from the rhizosphere must be removed and the scraping, flushing, and leaching technique is commonly used. But choosing appropriate cultural practices is the key to reducing the effect of salinity on the growth and yield of plants, whereas seed treatment with macro, micro, and plant growth regulators (PGRs) is a lively and short-term method of dealing with salinity stress (Zodape et al., 2011). Some pharmacological treatments, such as ascorbic acid (Vitamin C) (Farooq et al., 2010), proline, glycinebetaine (Abbas et al., 2010), silicon, and triacontanol (Ashraf et al., 2010) have been utilized in the past to regulate the condition. To counteract the yield loss caused by salt in eggplant, these strategies must be used, especially in the short term (using chitosan). Chitosan is a linear polysaccharide made from chitin. Insect skeletons, fungal cell walls, and crab shells all naturally contain it. In nature, after cellulose, it is the biopolymer that occurs most frequently. Due to its use as a foliar spray, bio-pesticide, organic fertilizer, growth booster, and other agricultural applications, chitosan has attracted increased attention (Górnik et al., 2008). According to Andres et al. (2007), it has biological properties that include being antibacterial, inhibiting the growth of some pathogens, immune system augmentation for plants, and stress relief from abiotic factors including salt, heat, heavy metal toxicity, and drought (Farouk et al., 2011).

Such methods can be used in the agricultural sector, especially for brief periods of time to manage sensitivity to salt stress. Pre-sowing seed treatment with chitosan can enhance salt tolerance in bitter gourd, a crop known for its sensitivity to salinity. This experiment, which was conducted to determine bitter gourd’s response to salinity tolerance and to determine how pre-sowing seed treatment with chitosan can lessen the lethal effect of salinity on bitter gourds, was carried out because of the importance of bitter gourds, the harmful effects of salinity, and the efficacy of chitosan.

## 2 Materials and methods

The study was conducted during the 2022 growing season at the Faculty of Agricultural Sciences (FAS), University of the Punjab, Lahore, Pakistan. Bitter gourd seeds, procured from a local market in Lahore, were subjected to various pre-sowing treatments to assess their response to salt stress. The experiment followed a completely randomized design (CRD) with seven treatments and four replications: T0 = Untreated seeds (non-saline), T1 = 50 mM NaCl (salt stress), T2 = hydroprimed seeds (50 mM NaCl), T3 = 50 mM NaCl + chitosan 0.01%, T4 = 50 mM NaCl + chitosan 0.02%, and T5 = 50 mM NaCl + chitosan 0.03%. The seeds were sown in plastic containers filled with sand, chosen for their well-defined properties in simulating saline stress. The sand had a pH ranging from 6.0 to 6.5, a field capacity of 7.2%, and incipient wilting of 1.2%, with Hoagland solution (0.5 strength) used as the feeding medium. Before priming treatments, seeds were soaked in a 5.0% NaOCl solution for 3 min to prevent fungal contamination (Ruan, 2002), followed by hydropriming and chitosan treatment for 16 hours. Chitosan (Sigma-Aldrich C3646, ≥75% deacetylated) was applied at concentrations of 0.01%, 0.02%, and 0.03%. After priming, the seeds were washed three times with double-distilled water (Khan et al., 2009). Sodium chloride (50 mM) was applied to the soil in the salt treatments (Sarwar et al., 2022) and the electrical conductivity (EC) of the sand was monitored throughout the experiment to assess the consistency of salinity levels. This experimental setup aimed to evaluate the effects of chitosan and salt stress on the morphological, physiological, and biochemical traits of bitter gourd under varying salt conditions.

### 2.1 Germination test

Using the methods mentioned by (Analysts, 1978), the possibility of bitter gourd seed germination was evaluated. Four replicates of each treatment were planted in 12 L pots with layers of moist sand between them at a temperature of 25°C. When root length increased to 2 mm, one seed was scored. Daily counts of all emerging seeds were made; observation began on the second day of ingestion and lasted 10 days.

### 2.2 Mean germination time (days)

In accordance with Ellis and Roberts’ (1981) technique, the mean emergence time (Ahmad et al., 2014) was calculated as follows:


$$\text{MGT}=\frac{\sum \text{Dn}}{\sum \text{n}}$$

(N= number of seed emerged on day D)

(D= number of days counted from beginning of test)

### 2.3 Germination index

The Association of Official Seed Analysts’ (1983) manual defines the procedure for measuring germination index (GI) and this method was followed for the calculation of GI.

### 2.4 Final germination percentage

The final bitter gourd seed emergence rate was recorded on day 15. It defined the percentage of seeds that germinated compared to the total seeds sowed.

### 2.5 Morphological attributes

Bitter gourd morphological parameters such as plant height and root and shoot dry biomass were measured and the average of all replicates were calculated (Sarwar et al., 2017).

### 2.6 Leaf chlorophyll contents (SPAD)

Portable equipment (Model: SPAD-502; Konica Minolta, Japan) was used to estimate the chlorophyll content of bitter gourd leaves at the crop maturity stage (Khan et al., 2003).

### 2.7 Electrolyte leakage (%)

Membrane leakage (Lutts et al., 1996) allows us to calculate the permeability of a membrane. Electrical conductivity measurement equipment (Mode: CC 501, Elmetron, Zabrze; Poland) was used. Before harvesting, ten discs of bitter gourd leaves were randomly chosen from five plants in each replication. These leaves were the newest, fully developed variety. These bitter gourd leaves were washed to get rid of any surface impurities before being placed in a test tube with 10 cc of distilled water. The solution’s electrochemical conductivity (EC1) was measured during a 24-hour incubation period at room temperature (25 2°C) on a shaker. These tubes were then placed in a water bath for 20 min, and once the solution had cooled, a second EC reading (EC2) was obtained. EL was calculated by this equation:


[EC1/EC2 and expressed into %]

### 2.8 Proline composition (μmol g^-1^ F.W)

A solution containing (2.0) ml of filtered homogenate of bitter gourd leaf samples, 2.0 ml of ninhydrin solution (1.25 g ninhydrin in 30.0 ml glacial acetic acid and 20.0 mL 6 Molar orthophosphoric acid), and 2.0 ml of glacial acetic acid that was heated at 100°C for 60 min was used to determine the proline contents according to the method of Bates et al., 1973. The reaction was subsequently ended by shifting the test tubes containing this mixture into an ice bath. The reaction mixture was then extracted with 10.0 ml of toluene while being vigorously agitated for 1-2 min through the passage of a constant air stream. Chromophore toluene was aspirated, and the aqueous phase was separated and warmed to room temperature. Using a double beam spectrophotometer with toluene as a blank, absorbance was measured at 520 nm. Proline contents were computed using fresh weight data and estimated using a standard curve (Model; Hitachi-120, Japan).


$$\begin{array}{c} {\text{Mole proline gm}}^{-1}\text{ fresh weigh}\\ =[{\text{gm proline ml}}^{-1}\times \text{ml of toluene}/115.5)/(\text{gm of sample}/5)] \end{array}$$

### 2.9 Anti-oxidant enzymes

Fresh samples of bitter gourd leaves (0.5g) were ground in an ice-cooled tissue grinder in (5.0 ml of 50.0 mM) cooled phosphate buffer pH 7.8 to determine the antioxidant activity. The homogenous mixture was centrifuged for 20 min at 15000 rpm and 4°C, and the supernatant was used to determine the following antioxidants’ activities.

#### 2.9.1 Superoxide dismutase (Unit’s/mg protein)

Superoxide dismutase activity was evaluated using the method of Giannopolitis and Ries (1977), which measures the ability of the enzyme to inhibit nitroblue tetrazolium (NBT) photoreduction. The reaction mixture (3.0 mL) consisted of 20–50 µL of enzyme extract, 50.0 mM NBT, 1.3 mM riboflavin, 13 mM methionine, 75.0 mM EDTA, and 50.0 mM phosphate buffer at pH 7.8. Test tubes containing the reaction mixture were exposed to light from 15 fluorescent lamps (78 µmol m-2 s^-1^) for 15 min. The absorbance of the solution was measured at 560 nm using a spectrophotometer (Model: Hitachi-650, Japan). One unit of SOD activity was defined as the amount of enzyme required to inhibit 50% of NBT photoreduction.

#### 2.9.2 Catalase and peroxidase (Unit’s/mg protein)

Catalase (CAT) and peroxidase (POD) activities were measured using the method described by Chance and Maehly (1955) with slight modifications. For the CAT assay, the reaction mixture (3.0 mL) contained 0.1 mL of enzyme extract, 5.9 mM H_2_O_2_, and 50.0 mM phosphate buffer at pH 7.0. Changes in absorbance were recorded every 20 sec at 240 nm. CAT activity was defined as a 0.01 change in absorbance per min. For the POD assay, the reaction solution (3.0 mL) consisted of 50.0 mM phosphate buffer (pH 5.0), 20.0 mM guaiacol, 40.0 mM H_2_O_2_, and 0.1 mL enzyme extract. Absorbance changes were measured at 470 nm every 20 sec. The enzyme activity for both CAT and POD was expressed as units per milligram of protein, with one unit of POD activity defined as a 0.01 change in absorbance per min (Hill et al., 2006).

### 2.10 Leaf water potential (-Ψw)

To evaluate the water potential, the third-youngest completely developed leaf from bitter gourd plants was chosen. Two of these leaves, one from each replication, were then cut at the end of the petiole using a sharp razor (Model: 615; USA). Before sunrise at 6 am, water potential values were recorded since sunlight triggers photosynthesis in plants, which begins water activity.

### 2.11 Leaf osmotic potential (- Ψs)

The exact bitter gourd leaf that was used to test the water potential was stored in a zipper bag and put in the freezer for 7 days at -80°C. After that, the frozen leaf was thawed at room temperature (25°C) for 30 min, and cell sap was extracted using a disposable syringe. Finally, 10 L of sap was applied with a syringe to the osmometer sensor to determine the osmotic potential, and data were recorded (Model: Wescor Model.; 5500).

### 2.12 Leaf turgor potential (Ψp)

The difference between the water potential and osmotic potential was used to calculate the bitter gourd leaf’s turgor potential. Thus, the following formula was used to calculate turgor potential:


(Ψp=Ψw–Ψs)

### 2.13 Leaf relative water contents

Each replication plant had three mature bitter gourd leaves that were cut off with a sharp razor and marked. The leaves were then cleaned with tap water for at least 5 min and dried using tissue paper. Each leaf was blotted individually, and then its weight was determined after being immersed in distilled water for a period of time (24 hours). After that, the leaves of the bitter gourd were dried in an oven at 72°C, and the dry weight of each leaf was determined using the method of Barrett-Lennard (2002).

{LFW=leaf fresh weight}

{LDW=leaf dry weight}

{LTW=leaf turgid weight}


{LRWC(%)=[(LFW−LDW)/(LTW−LDW)]×100}

### 2.14 Measurement of ionic status

#### 2.14.1 Determination of Na*^+^*, K*^+^*, and Ca^+^ (mg g^-1^ D.W)

Wolf’s (1990) method was used for the determination of Na^+^ and K^+^ in the plants. Bitter gourd leaf samples that had been digested were tested for Na^+^, K^+^, and Ca^+^ (Flame photometer model: Jenway PFP-7, UK). The values of Na^+^, Ca^+^, and K^+^ as measured by a flame photometer were compared with the standard curve and the original quantities were compared. A graded series of standards (ranging from 20 to 100 mg L^-1^) of Na^+^, Ca^+^, and K^+^ were made.

#### 2.14.2 Chloride (Cl^-^) determination (mg g^-1^ D.W)

Bitter gourd leaves were ground into a fine powder using a grinder, and then the powdered plant material (1.0 g) was heated with distilled water (20 ml) in test tubes at 65°C for an overnight period. After being heated for the entire night, the extract was filtered through Whatmann 40 filter paper and used to calculate the number of chloride ions in the sample using a chloride analyzer (Model: Corning-920; Germany).

### 2.15 Statistical analysis

Statistix 8.1 was used to calculate the analysis of variance and multiple comparison test (Tukey’s HSD test). Differences between treatments were determined to be significant after statistical analysis at P ≤0.05 (Stein et al., 1997).

## 3 Results

### 3.1 Effect of seed treatment with chitosan on mean germination time of bitter gourd plants under saline conditions

The collected data of mean germination time (MGT, Days) were subjected to analysis of variance and it revealed a significant result for all studied treatments. It can be concluded from the study that the mean emergence time (MET, days) decreased in the chitosan-treated seeds under salt stress. The least time for germination was observed in T_6_ = Saline (50 mM NaCl) + chitosan 0.04%. The seeds under control conditions (non-saline + un-treated seeds) had the highest MET whereas seeds that were hydroprimed exhibited insignificant differences from the control. Chitosan-treated seeds i.e., T_6_ = Saline 50 mM + chitosan 0.04% had the lowest MET. **Table 1** shows that the bitter gourd plants grown under T_1_ = 50 mM saline stress took the longest time for emergence. Overall, chitosan at 0.04% was observed to be the best dose for early germination as compared to the control.

**Table 1:** Effect of seed treatment with chitosan on mean germination time (MGT), germination index (GI) and final germination percentage (FGP) of bitter gourd plants under saline conditions.

Treatments	MGT	GI	FGP
T_0_	8.2225 ± 8.99^bc^	7.660 ± 4.88^cd^	67.200 ± 1.81^ab^
T_1_	8.9525 ± 4.93^a^	4.103 ± 2.52^e^	37.065 ± 1.97^d^
T_2_	8.3450 ± 3.90^b^	6.965 ± 3.44^d^	54.712 ± 2.35^c^
T_3_	8.0725 ± 4.99^bcd^	8.730 ± 1.72^bc^	64.585 ± 1.12^b^
T_4_	7.9950 ± 3.23^cd^	9.140 ± 1.64^b^	66.220 ± 1.18^ab^
T_5_	8.1475 ± 9.51^bc^	9.053 ± 3.86^bc^	67.865 ± 1.61^ab^
T_6_	7.7200 ± 4.32^e^	10.860 ± 3.76^a^	72.215 ± 1.60^a^
T_7_	7.8375 ± 5.00^de^	9.405 ± 3.19^b^	65.120 ± 1.51^ab^

Data represent mean ± SE, followed by different letters indicate significant differences as per Tukey’s HSD test (P ≤ 0.05).

### 3.2 Effect of seed treatment with chitosan on germination index of bitter gourd plants under saline conditions

The finding from the statistical analysis showed that the GI was significantly affected in the chitosan-treated seeds. Graphical bars show that the highest germination index was noted in chitosan-treated seeds under saline conditions. Hydroprimed seeds revealed a slight improvement, followed by the control. Under salinity 50 mM, chitosan treatment T_6_ = S + chitosan 0.04% shown better performance as compared to the control (**Table 1**). The lowermost GI was exhibited by T_0_, un-treated seeds, and T_1_ = 50 mM salt stress.

### 3.3 Effect of seed treatment with chitosan on final germination percentage of bitter gourd plants under saline conditions

The analysis of variance regarding final germination percentage of bitter gourd plants under saline conditions showed highly significant effects for all treatments. The results revealed a reduction in final germination percentage in plants under salt stress when compared with control plants and an improvement in final germination percentage of seeds treated with different doses of chitosan. The highest final germination percentage (72.215%) was recorded in T_6_ = S + chitosan 0.04% treated seeds. Whereas, the lowest final germination percentage (37%) was observed in the T_1_ = 50 mM saline stress treatment. The seeds that were hydroprimed showed less improvement in final germination percentage compared those seeds treated with chitosan as shown in **Table 1**.

### 3.4 Effect of seed treatment with chitosan on morphological parameters of bitter gourd plants under saline conditions

Seed treatment with chitosan significantly improved various morphological parameters of bitter gourd plants under saline conditions (**Table 2**). The highest shoot length (77.17 cm) was observed in the control plants, while salinity stress (50 mM) reduced it to 52.85 cm. However, chitosan treatment (T6: S + chitosan 0.04%) resulted in a 23.84% increase in shoot length compared to salinity-stressed plants. Similarly, root length, which was highest in control plants (38.70 cm), decreased under salinity stress (29.85 cm). Chitosan treatment, particularly at 0.04% (T6), improved root length significantly over salinity-stressed plants. Regarding fresh weight, chitosan-treated plants (T6) exhibited a 65.48% increase in plant fresh weight (44.05 g) compared to salinity-treated plants (26.62 g). The highest dry weight (17.55 g) was found in control plants, while salinity stress reduced it to 8.91 g. Chitosan treatment (0.04%) resulted in a 71.21% increase in dry weight over the salinity-only treatment. Overall, chitosan priming enhanced plant growth and mitigated the negative effects of salinity stress, with 0.04% chitosan showing the most effective results.

**Table 2:** Effect of seed treatment with chitosan on morphological parameters of bitter gourd plants under saline conditions.

Treatment	Shoot length (cm)	Root length (cm)	Plant fresh weight (g)	Plant dry weight (g)
T_0_	77.175 ± 3.58^a^	38.700 ± 8.66^a^	50.050 ± 3.25^a^	17.550 ± 1.96^a^
T_1_	52.850 ± 3.04^b^	29.850 ± 1.23^b^	26.620 ± 1.77^d^	8.907 ± 8.35^c^
T_2_	56.700 ± 3.32^b^	31.050 ± 7.5^b^	30.167 ± 7.35^cd^	9.750 ± 1.10^bc^
T_3_	60.007 ± 1.96^b^	31.200 ± 1.76^b^	35.585 ± 2.56^bcd^	11.853 ± 8.31^bc^
T_4_	62.335 ± 3.09^b^	32.950 ± 2.04^ab^	37.840 ± 2.99^abcd^	12.367 ± 1.70^abc^
T_5_	64.125 ± 2.24^ab^	33.450 ± 0.75^ab^	40.783 ± 1.50^abc^	14.015 ± 1.98^abc^
T_6_	65.450 ± 2.09^ab^	35.850 ± 1.02^ab^	44.050 ± 2.27^ab^	15.250 ± 8.33^ab^
T_7_	62.352 ± 5.76^b^	33.200 ± 1.88^ab^	38.610 ± 5.60^abcd^	12.375 ± 1.07^abc^

Data represented as Mean ± SE, and different letters indicate significant differences as per Tukey’s HSD test (P ≤ 0.05).

### 3.5 Effect of seed treatment with chitosan on chlorophyll contents (SPAD) of bitter gourd plants under saline conditions

**Figure 1:**
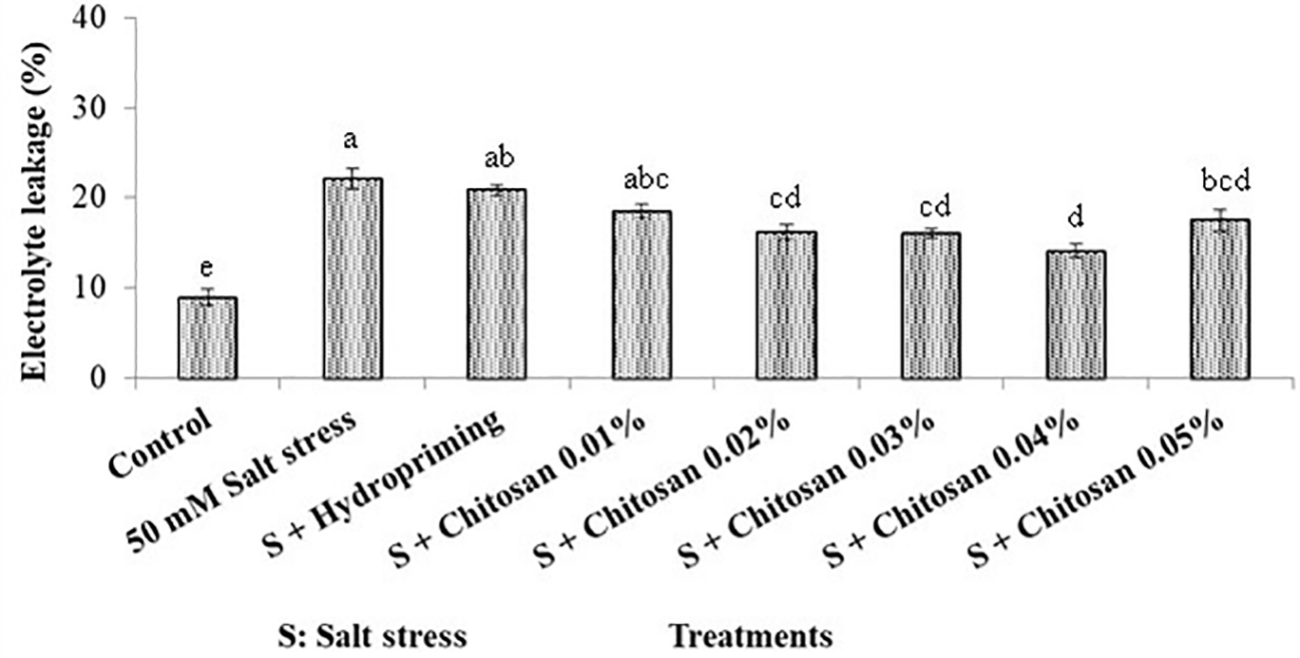
Effect of seed treatment with chitosan on chlorophyll contents (SPAD) of bitter gourd plants under saline conditions. Data represented as Mean ± SE and different letters on the top of the bars indicate significant differences as per Tukey’s HSD test (P ≤ 0.05).

Analysis of variance of the data regarding chlorophyll contents of bitter gourd plants treated with chitosan under saline conditions showed highly significant results of treatment effect. As shown in **Figure 1**, the highest chlorophyll contents (29.5 SPAD) were noticed in the control plants and lowest (22.5 SPAD) in those plants that were under salinity stress (T_1_ = 50 mM). However, the seed treatments with chitosan did not show any significant improvement in chlorophyll contents except for the T_6_ (S + chitosan 0.04%) treatment. The total chlorophyll contents in T6 plants were 26.250 SPAD and it led to an improvement of 16.667% in this attribute as compared to plants treated with T_1_ (50 mM salinity stress).

### 3.6 Effect of seed treatment with chitosan on electrolyte leakage (%) of bitter gourd plants under saline conditions

**Figure 2:**
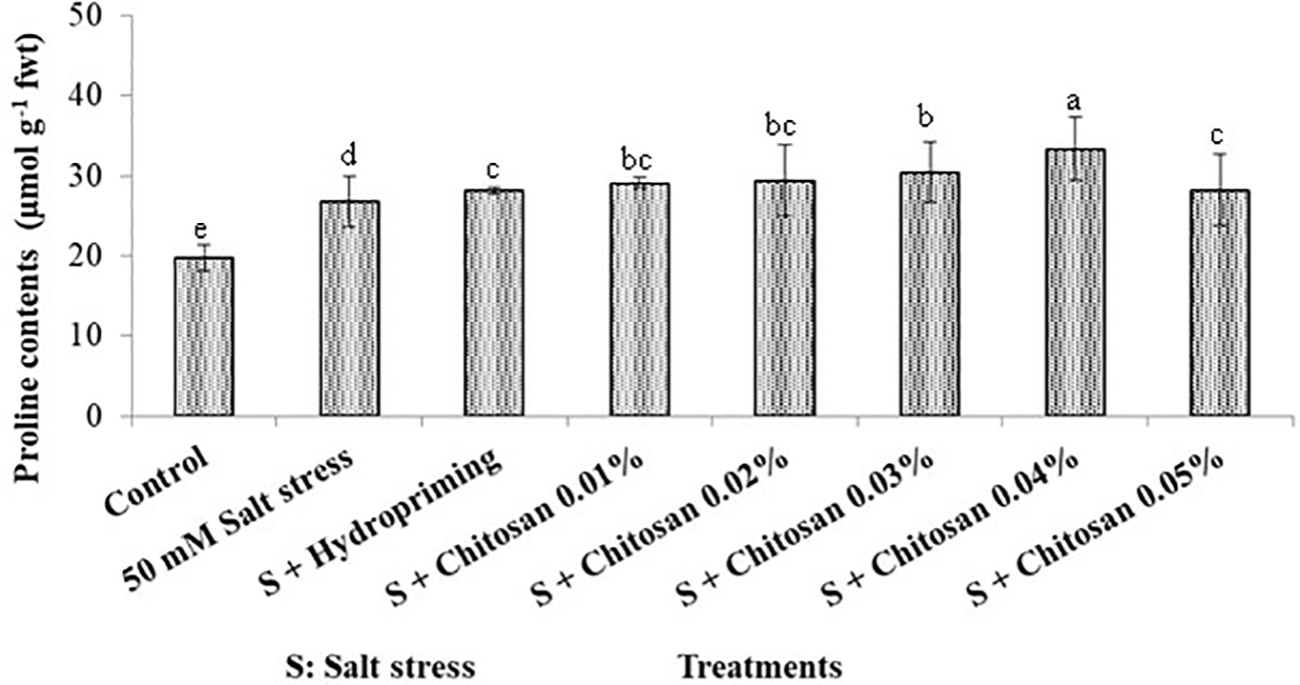
Effect of seed treatment with chitosan on electrolyte leakage (%) of bitter gourd plants under saline conditions. Data represented as Mean ± SE and different letters on the top of bars indicate significant differences as per Tukey’s HSD test (P ≤ 0.05).

**Figure 2** shows that the highest electrolyte leakage (22.093%) was noticed in plants exposed to salinity stress without any seed priming (T_1_). The priming of seeds with chitosan assisted the plants to survive the adverse effect of salinity and reduced the electrolyte leakage up to 36.006% in the T_6_ (S + chitosan 0.04%) treated plants as compared to plants treated with T1 = 50 mM salinity stress alone. However, the higher dose of chitosan (T_7_= S + chitosan 0.05%) was less effective than T_6_= S + chitosan 0.04%, T_5_= S + chitosan 0.03%, and T4= S + chitosan 0.02% for this attribute. Furthermore, the seeds that were hydroprimed showed only a 5.567% reduction in electrolyte leakage as compared to the T_1_-treated plants.

### 3.7 Effect of seed treatment with chitosan on proline contents (µmol g^-1^ fwt) of bitter gourd plants under saline conditions

**Figure 3:**
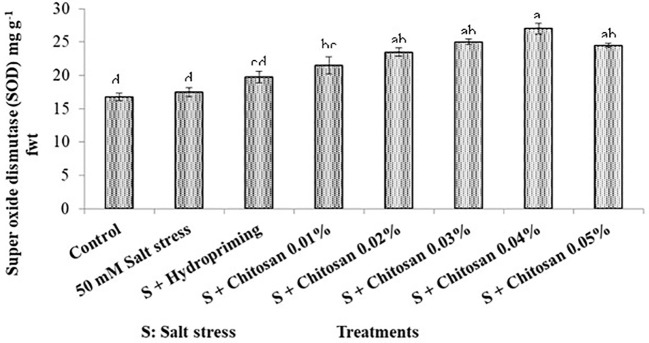
Effect of seed treatment with chitosan on proline contents (µmol g^-1^ fwt) of bitter gourd plants under saline conditions. Data represented as Mean ± SE and different letters on the top of bars indicate significant differences as per Tukey’s HSD test (P ≤ 0.05).

The graphical bars in **Figure 3** show that the proline contents (mean) of bitter gourd was as follows: T_6_ (33.350 µmol g^-1^ fwt) > T_5_ (30.375 µmol g^-1^ fwt) > T_4_ (29.405 µmol g^-1^ fwt) > T_3_ (29.073 µmol g^-1^ fwt) > T_7_ (28.147 µmol g^-1^ fwt) > T_2_ (28.100 µmol g^-1^ fwt) > T_1_ (26.800 µmol g^-1^ fwt) > T_0_ (19.775 µmol g^-1^ fwt). The seed priming with chitosan caused the highest increase (24.440%) in plant proline contents at T_6_= S + chitosan 0.04% as compared to plants treated with salinity alone (T_1_). However, this increase in proline contents was statistically insignificant.

### 3.8 Effect of seed treatment with chitosan on SOD in bitter gourd plants under saline conditions

**Figure 4:**
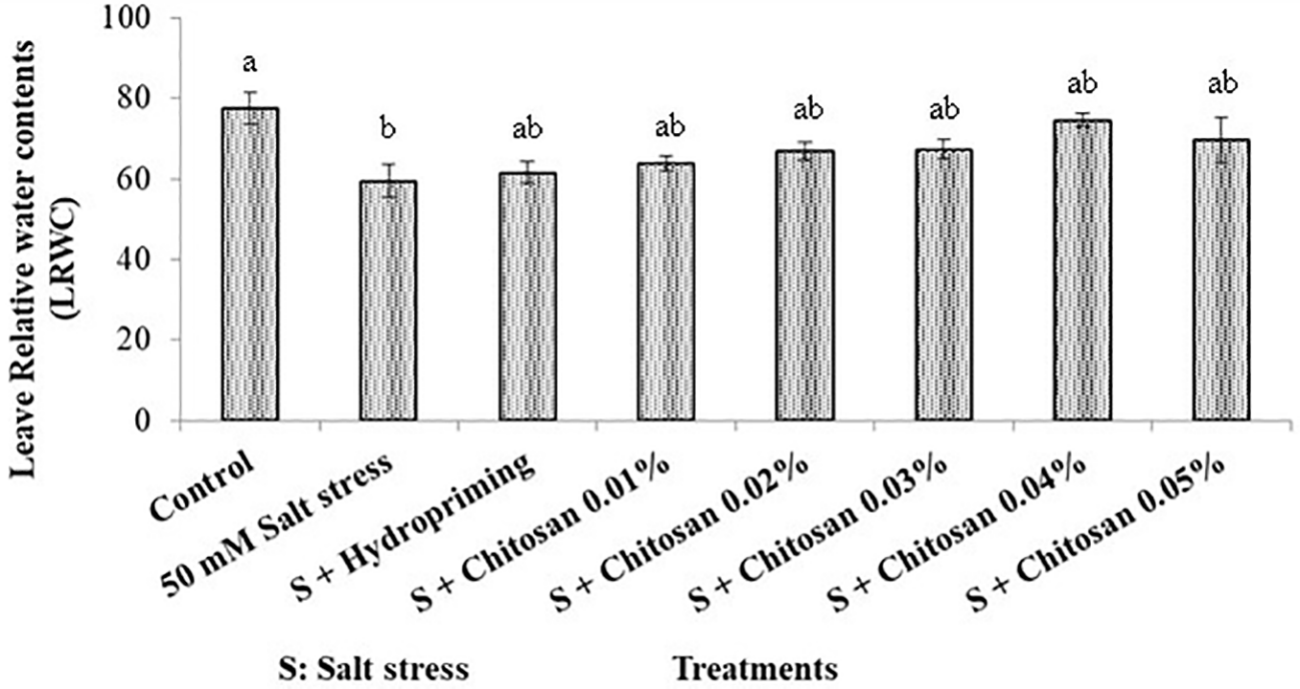
Effect of seed treatment with chitosan on super oxide dismutase (mg g^-1^) of bitter gourd plants under saline conditions. Data represented as Mean ± SE and different letters on the top of bars indicate significant differences as per Tukey’s HSD test (P ≤ 0.05).

The results indicated that the highest superoxide dismutase activity (27.000 mg g^-1^ fwt) was found in the T6 (S + chitosan 0.04%) plants and the least (16.807 mg g^-1^ fwt) in the T0 (control) plants. However, the superoxide dismutase activity of plants treated with salinity stress alone (T1 = 50 mM salt stress) was recorded as 17.500 mg g^-1^ fwt. The seed priming of bitter gourd with chitosan increased the superoxide dismutase activity up to 54.285% in T6 (S + chitosan 0.04%) as compared to plants treated with salinity stress alone (**Figure 4**).

### 3.9 Effect of seed treatment with chitosan on POD in bitter gourd plant under saline conditions

The results indicated the highest peroxide activity (8.087 mg g^-1^ fwt.) in the T_6_ (S + chitosan 0.04%) treatment and the least (5.397 mg g^-1^ fwt.) in the control plants. The plants treated with only 50 mM salinity stress were found to have 6.145 mg g^-1^ peroxidase activity. The effect of treatments T_3_, T_4_ and T_7_ did not vary significantly from each other for this attribute. It is apparent from the results that the seed priming with chitosan increased the peroxide activity of bitter gourd and it was the highest at the 0.04% chitosan dose. The seed priming with chitosan 0.04% increased peroxidase activity by up to 49.842%, 31.602%, and 26.458% compared with the control, T1, and hydroprimed seeds, respectively (**Table 3**).

**Table 3:** Effect of seed treatment with chitosan on peroxidase (POD), catalase (CAT), leaf osmotic potential (Méndez-Bautista et al.) and leaf water potential (LWP) in bitter gourd plants under saline conditions.

Treatments	Peroxidase	Catalase	LOP	LWP
T_0_	5.397 ± 4.41^c^	0.357 ± 1.37^e^	0.7185 ± 3.99^d^	0.4450 ± 3.93^c^
T_1_	6.145 ± 0.31^bc^	0.412 ± 0.01^de^	1.0817 ± 4.22^a^	0.7275 ± 5.21^a^
T_2_	6.395 ± 3.47^abc^	0.430 ± 2.48^cde^	0.9938 ± 2.69^ab^	0.7025 ± 5.84^a^
T_3_	6.645 ± 1.44^abc^	0.457 ± 1.88^b-e^	0.9425 ± 1.10^abc^	0.6950 ± 2.10^a^
T_4_	6.895 ± 5.09^abc^	0.502 ± 0.01^bcd^	0.9200 ± 2.73^bc^	0.6350 ± 2.36^ab^
T_5_	7.395 ± 2.46^ab^	0.547 ± 2.49^ab^	0.8650 ± 0.25^bcd^	0.5700 ± 4.08^abc^
T_6_	8.087 ± 3.43^a^	0.627 ± 2.78^a^	0.8050 ± 3.36^cd^	0.4575 ± 4.53^bc^
T_7_	6.517 ± 5.06^abc^	0.545 ± 4.29^abc^	0.8295 ± 2.12^cd^	0.5775 ± 0.03^abc^

Data represented as Mean ± SE and different letters indicate significant differences as per Tukey’s HSD test (P ≤ 0.05).

### 3.10 Effect of seed treatment with chitosan on CAT in bitter gourd plants under saline conditions

The highest catalase activity (0.627 mg g^-1^) of bitter gourd was recorded in the T_6_ (S + chitosan 0.04%) treated plants and the least (0.357 mg g^-1^ fwt.) in the control plants. The plants under 50 mM salt stress and without any seed treatment were found to have 0.412 mg g^-1^ catalase activity. The comparison of the control plants with the T_1_-treated plants showed a difference of 15.406% in catalase activity and the latter was higher than the former. However, the seed priming with 0.04% chitosan further increased the catalase activity by 52.305% compared with the T_1_-treated plants (**Table 3**).

### 3.11 Effect of seed treatment with chitosan on leaf osmotic potential in bitter gourd plants under saline conditions

The interpretation of statistical data regarding the osmotic potential (Ψs) of bitter gourd plants treated with chitosan under saline conditions showed highly significant differences among treatments. All the treatment means also varied significantly from the control (**Table 3**).

**Table 3** shows that the highest osmotic potential (-1.081 MPa) was found in the T_1_ (50 mM salinity stress) plants and the least (-0.7185 MPa) in the control plants. However, the seed priming of bitter gourd with chitosan showed a reduction in osmotic potential of up to 25.580% in T_6_ (S + chitosan 0.04%), 23.315% in T_7_ (S + chitosan 0.05%), 20.033% in T_5_ (S + chitosan 0.03%), 14.948% in T_4_ (S + chitosan 0.02%), 12.868% in T_3_ (S + chitosan 0.01%), and 8.126% in the hydroprimed seeds (T_2_) compared to plants under salt stress only (T1 = 50 mM Salt Stress).

### 3.12 Effect of seed treatment with chitosan on leaf water potential in bitter gourd plants under saline conditions

The highest leaf water potential (-0.727 MPa) in bitter gourd was recorded in the T_1_ (50 Mm salt stress) treated plants and the least (-0.445 MPa) in the control plants.

The comparison of the control plants with T_1_-treated plants showed that salinity stress caused an increase of 68.483% in the leaf water potential in bitter gourd plants. However, the seed priming with chitosan reduced the impact of salt stress up to 37.113% in the T_6_ (S + chitosan 0.04%) plants regarding this attribute. Hydropriming only reduced the salinity impact up to 3.436% on leaf water potential in bitter gourd (**Table 3**).

### 3.13 Effect of seed treatment with chitosan on leaf relative water contents in bitter gourd plants under saline conditions

**Figure 5:**
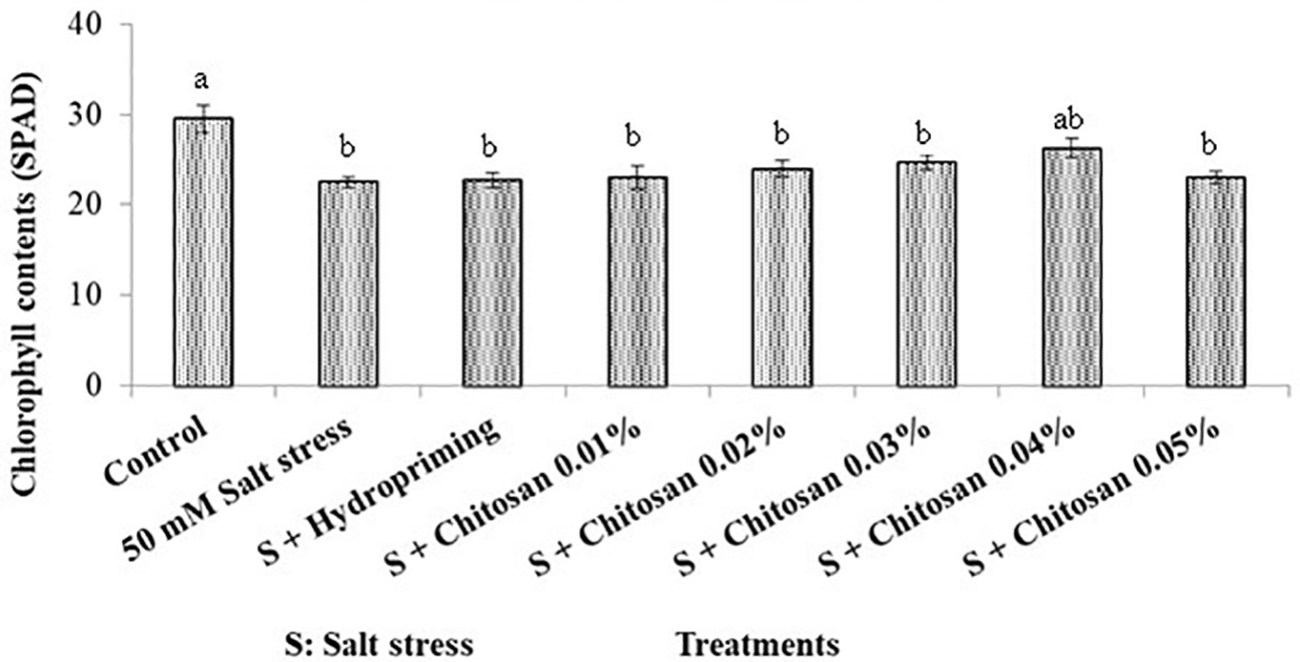
Effect of seed treatment with chitosan on leaf relative water contents in bitter gourd plants under saline conditions. Data represented as Mean ± SE and different letters on the top of bars indicate significant differences as per Tukey’s HSD test (P ≤ 0.05).

The highest leaf relative water contents (77.500%) in bitter gourd was recorded in control plants and least (59.500%) in T_1_ (50 Mm Salt Stress) treated plants. The comparison of hydropriming and seed priming with chitosan treatments showed non-significant difference among these treatments for this parameter. The leaf relative water contents in T_2_ (S +Hydropriming), T_3_ (S + Chitosan 0.01%), T_4_ (S + Chitosan 0.02%), T5 (S + Chitosan 0.03%), T_6_ (S + Chitosan 0.04%), and T_7_ (S + Chitosan 0.05%) treated bitter gourd plants was recorded as 61.525%, 63.902%, 66.905%, 67.375%, 74.500% and 69.660% respectively. Furthermore, it is evident from results that 0.04% chitosan caused the maximum increase (25.210%) in leaf relative water contents of bitter gourd plant under salinity stress but it is still 3.871% less than the control plants (**Figure 5**).

### 3.14 Effect of seed treatment with chitosan on Na^+^ content in bitter gourd plants under saline conditions

From the results, the highest Na^+^ content (23.055 mg g^-1^ dry wt.) was noticed in the T_1_ (50mM Salt Stress) treated plants and the lowest (8.813 mg g^-1^ dry wt.) in the control plants. Salinity stress caused an increase of 161.602% in Na^+^ content in bitter gourd plants. However, seed treatment with chitosan under salinity stress reduced the effect of stress and caused a reduction in Na^+^ content up to 31.25% in the T_6_ (S + Chitosan 0.04%) treated plants compared to the T_1_ (50mM Salt Stress) treated plants. Furthermore, hydropriming only reduced the Na^+^ content up to 3.253% in bitter gourd plants under salt stress (**Table 4**).

**Table 4:** Effect of seed treatment with chitosan on Na^+^, K^+^, leaf osmotic potential (Méndez-Bautista et al.), and leaf water potential (LWP) of bitter gourd plants under saline conditions.

Treatments	N^+^	K^+^	Ca^+^	Cl^-^
T_0_	8.813 ± 6.12^d^	37.250 ± 1.93^a^	39.500 ± 2.10^a^	6.510 ± 5.36^b^
T_1_	23.055 ± 8.58^a^	20.500 ± 2.01^b^	22.500 ± 1.55^b^	12.665 ± 1.24^a^
T_2_	22.305 ± 1.51^ab^	21.850 ± 2.26^b^	22.947 ± 2.38^b^	11.948 ± 1.18^a^
T_3_	20.073 ± 1.02^abc^	23.072 ± 2.35^b^	23.115 ± 2.17^b^	10.865 ± 1.35^ab^
T_4_	18.155 ± 7.27^bc^	25.655 ± 4.35^ab^	26.003 ± 1.64^b^	10.003 ± 8.25^ab^
T_5_	18.125 ± 8.29^bc^	26.375 ± 3.95^ab^	28.615 ± 1.29^b^	9.615 ± 1.67^ab^
T_6_	15.850 ± 1.16^c^	28.850 ± 2.92^ab^	30.000 ± 1.49^ab^	8.987 ± 6.05^ab^
T_7_	17.365 ± 7.95^c^	23.390 ± 2.24^b^	28.557 ± 2.85^b^	10.420 ± 1.20^ab^

Data represented as Mean ± SE and different letters indicate significant differences as per Tukey’s HSD test (P ≤ 0.05).

### 3.15 Effect of seed treatment with chitosan on K^+^ content in bitter gourd plants under saline conditions

**Table 4** indicates that the highest K^+^ content (37.250 mg g^-1^ dry wt.) was found in the control plants and the least (20.50 mg g^-1^ dry wt.) in the T1 (50 mM salinity stress) treatment. However, the seed priming of bitter gourd with chitosan showed an improvement in the K^+^ contents compared to plants treated with salinity stress alone. The highest increase (28.850 mg g^-1^ dry wt.) caused by chitosan was noted in T_6_= S + chitosan 0.04% compared to T_1_. Furthermore, the seeds that were hydroprimed (T_1_) and primed with 0.01% or 0.05% chitosan did not vary significantly in K^+^ content from the T_1_ (50 mM salinity stress) treated plants.

### 3.16 Effect of seed treatment with chitosan on Ca^+^ content in bitter gourd plants under saline conditions

**Table 4** shows that the plant Ca^+^ content was highest (39.500 mg g^-1^ dry wt.) in the control plants and the least (22.500 mg g^-1^ dry wt.) in the T_1_ (50mM Salt Stress) treated plants. Salt stress caused a 43.038% reduction in the Ca^+^ content of the treated plants as compared to the control. The seed treatment with chitosan and hydropriming did not significantly improve the uptake of Ca^+^ content in bitter gourd plants except for the 0.04% chitosan treatment. It (S + chitosan 0.04%) caused an improvement of 33.333% in the Ca^+^ content of bitter gourd.

### 3.17 Effect of seed treatment with chitosan on Cl^-^ content in bitter gourd plants under saline conditions

From the results, it is evident that the highest Cl^-^ contents (12.665 mg g^-1^ dry wt.) was found in those plants that were under salinity stress (T_1_ = 50 mM) and the lowest (6.510 mg g^-1^ dry wt.) in the control plants (T_0_). Salinity stress caused an increase of 94.546% in Cl^-^ content in bitter gourd due to the uptake of NaCl. However, the seed treatment with chitosan helped the plants to handle the problem of salinity and reduced the uptake of Cl^-^ content up to 29.040% in the T_6_ (S + chitosan 0.04%) treated as compared to the T_1_ (50mM salt stress) treated plants. Furthermore, the effect of different doses of chitosan treatments did not differ significantly for this attribute. Conversely, the seeds that were hydroprimed (T_2_) did not show any significant difference in Cl^-^ content in bitter gourd plant compared with the T_1_ (50mM Salt Stress) treated plants (**Table 4**).

## 4 Discussion

Salinity is a factor in the global lack of land; 7% of the earth’s surface has salinized soils, but the extent of soils affected by Na^+^ is significantly greater. According to data from Pessarakli (1999), over 100 million hectares of arable land have been converted into salt-affected soils as a result of saline water, making up approximately 11% of all irrigated areas worldwide. It poses a serious threat to agricultural production in the most populous and financially struggling nations, including Pakistan, where it covers between 3 and 6 million hectares (Vashev et al., 2010), Bangladesh, where it covers more than 1 million hectares (Hossain et al., 2010), and India, where it covers more than 7 million hectares. A lack of nutrients, ionic imbalance, and the generation of reactive oxygen species (ROS) are all effects of salt stress. Ca^+^ ions replace Na^+^ ions in membranes, causing bridges that prevent the creation of proteins, enzymes, and nucleic acids (Pattanagul and Thitisaksakul, 2008). The harmful effects of saline stress are being reduced using a variety of approaches.

The use of plant growth regulator/promoting rhizobacteria (PGPR), grafting of vegetables, exogenous foliar spray of biostimulants/antioxidants/phytohormones/osmoprotectants, and seed priming are some examples of these procedures (Mavi, 2014). Chitosan has a significant role to play in the development of abiotic stress tolerance among all the others. The seeds in the current study were treated with chitosan at varying amounts when they were under salt stress. After data analysis, it was discovered that seeds treated with chitosan displayed a notable tolerance to salt stress by enhancing bitter gourd germination and growth. The first stage of plant morphology, seed emergence, is significantly impacted by salinization in the root zone. In essence, the presence of salt in the root zone results in high osmotic pressure, which ultimately causes cell dehydration. It also causes a high concentration of Na^+^ and Cl^-^ ions to build up in the soil solution, which interferes with the availability of nutrients, especially K^+^ ions (Hasanuzzaman et al., 2013). The highest concentration of Na^+^ and Cl^-^ ions in the root zone causes an ionic imbalance that limits the embryo’s ability to absorb water and causes the plumule and radicle tissues to collapse, which inhibits radicle growth and delays the emergence of the seeds (Maksimovic and Ilin, 2012; Gao et al., 2015). The results of this study make it abundantly evident that salt stress has a negative impact on bitter gourd seedling emergence and uniformity. Although chitosan-treated seeds take less time to germinate, they have an increased germination index and ultimate emergence percentage and demonstrated a significant improvement as the fatal effects of salt stress were reduced. Previous research demonstrated that chitosan significantly increased the rate of germination in cucumber and brinjal crops (Ali et al., 2007). The consistent crop emergence caused by seed treatment may be attributable to the stimulation of numerous biochemical processes in the seeds, such as dormancy breaking, hydrolysis, and enzyme activation that initiate the germination process (Ali et al., 2007). Beginning seed germination with few variations is a crucial indicator of crop uniformity and seedling vigor. Conversely, crops are deemed to be more vigorous if they emerge fully within a short period of time. The results showed greater improvement in morphological parameters, such as plant height, root length, and root and shoot fresh and dry weights, under stressed conditions. Chitosan-treated plants emerge early and grow well. Ma et al. (2011) confirmed the current findings and claimed that chitosan treatments improved wheat crop growth under salt stress. Chitosan supports the regulation of plant growth, development, and morphogenesis processes (Côté and Hahn, 1994). Additionally, it activates and speeds up plant defense mechanisms by increasing the activity of certain enzymes, such as pectinases, glucanases, and chitinases, which promote the growth of eggplant (Hien, 2004). Chitosan increases the availability of water and nutrients by changing the osmotic potential of cells under salt stress (Guan et al., 2009). Although abiotic factors such as salt have a negative impact on plant growth, plant height is solely determined by genetics, as shown by the findings of the current study. Without chitosan seed treatments, salt stress may cause nutritional deficiency, metabolic pathway disruptions, and ion toxicity in plants, which may all contribute to their poor performance. By reducing the activity of photosynthetic enzymes, salt stress also harms the pigments involved in photosynthesis (Ma et al, 2011). Under saline stress in bitter gourd, primed seeds’ chlorophyll levels were similarly increased. This increase in chlorophyll content in seeds treated with chitosan may be attributed to the protein complexes’ stability and chlorophyll’s protection from oxidation by the chlorophyllase enzyme. The results of this study revealed that under stress, treating seeds with chitosan had a favorable effect on the chlorophyll concentrations in bitter gourd plant leaves. The T_6_ therapy with chitosan was therefore found to be more responsive than all other therapies. According to Shoresh et al. (2011), the increase of harmful ions into plant leaves is connected with the loss in chlorophyll concentrations in eggplant under salt stress. Electrolyte leakage (%) indicates the stability of the membrane and indicates the level of oxidative stress in plants (Shi et al., 2015). Our findings showed that electrolyte leakage (%) increased under salt stress; this is an indication of ROS damage brought on by oxidative stress. Chitosan primed plants showed a significant decrease in electrolyte leakage (%) of leaves during saline stress. By reducing electrolyte leakage (%) in stressed plants, seed treatment with chitosan has produced positive outcomes; this may be because of the antioxidant enzymes that are produced in response to chitosan application. It appears that seed treatment with chitosan has a protective effect against membrane damage caused by salt. As a result, the current data pointed to an improvement in bitter gourd plant growth.

Proline concentrations were higher in the leaves of salt-stressed plants compared to controls; this may be because proline biosynthesis was induced, proline oxidation to glutamate was reduced, or proline was consumed less during protein synthesis (Procházková et al., 2016). While using chitosan as a seed treatment under salt stress resulted in a considerable rise in proline levels. According to (Iqbal and Ashraf, 2005), proline acts as a source of nitrogen to save plants in stressful situations by reducing their osmotic potential and uptake of deadly ions (Terzi et al., 2015). Our results also showed that the proline content under salinity was improved by chitosan compared to the control. Therefore, by reducing osmotic stress, it can be said that chitosan indirectly aided plant growth and development. This osmolyte performs various other tasks than osmotic adjustment, including water uptake, nutrition balancing, and cell turgor and integrity preservation. As a result, the above-mentioned factors may account for the tolerant eggplant genotype’s effective reaction in the current investigation. Chitosan increased the proline content of salinized tomato plants. It is possible that chitosan’s ability to reduce stress results from its beneficial effects on osmolytes, which let the plant make effective osmotic adjustments in stressful conditions.

Our findings showed that under salt stress, chitosan-treated plants had higher SOD activity than the control. High levels of ROS may increase the expression of genes that produce SOD, which in turn leads to higher SOD activity (Xing et al., 2015). The highest SOD activity under salt stress was observed to be associated with plants’ tolerance due to its ability to detoxify the superoxide radical (Ahmad et al., 2015). Other enzymes for removing H2O2 from cells are POD and CAT (Vuleta et al., 2015). In the present investigation with chitosan seed treatments under saline stress, both CAT and POD demonstrated a similar level of activity. Chitosan was therefore very effective at reducing the negative effects of salt on eggplant. These findings were in line with those of Lianju et al. et al (2012), who reported that wheat treated with chitosan and cultivated under salt stress showed an increase in SOD, POD, and CAT activities.

As compared to control, bitter gourd plants under stress had significantly lower leaf water potential, osmotic potential, turgor potential, and relative water contents. Chitosan seed treatments aided in the osmotic adjustment of salinized plants by causing a greater decrease in the Ψs and Ψw in bitter gourd. Our research has shown that chitosan may increase the osmotic adjustment mechanism by increasing organic osmolytes and decreasing the buildup of inorganic harmful ions. The findings of this study supported those of Tester and Davenport (2003), who hypothesized that inorganic ion concentrations such as Na^+^ and K^+^ are related to eggplant’s osmotic adjustment and salt tolerance. According to Assaha et al. (2013), similar kinds of observations have been made. The LRWC was considerably reduced by salt stress. These results are in agreement with Hegazi et al. (2015). LRWC was significantly improved by the chitosan treatment under saline stress. Chitosan also raised the amount of K^+^ in the leaf, which activated enzymes, caused stoma movement, polarized membranes, controlled osmotic pressure, and ultimately balanced osmotic and turgor potential. According to Farouk et al. (2011), chitosan increased the LRWC of radish under cadmium exposure.

In this work, salinity stress lowered the Ca^2+^ and K^+^ levels of the leaves while increasing the Na^+^ and Cl^-^ content. Beneficial nutrients, such as K^+^ and Ca^2+^, are particularly helpful for a plant’s growth and development because they control how proteins are made, enhance enzyme activity, and maintain the integrity of the plasma membrane and cell walls (Chen et al., 2012). In saline conditions, a significant effect was observed for leaf Na^+^, Cl^-^, K^+^, and Ca^2+^. A lack of chitosan resulted in poor growth due to a high accumulation of toxic ions (Na^+^ and Cl^-^) and low content of K^+^ and Ca^2+^ in the leaf, whereas the T6 treatment resulted in better growth due to low ratios of these toxic ions and higher amounts of K^+^ and Ca^2+^ in bitter gourd leaves. Toxic ion concentrations (Na^+^ and Cl^-^) in leaf tissues and plant growth are negatively correlated so poor growth can be seen. According to Guan et al. (2009) chitosan promotes plant development by increasing the uptake of water and the vital nutrients K^+^ and Ca^2+^ through changes in cell osmotic pressure.

## 5 Conclusion

It can be concluded from the present research that the optimized dose of 0.04% chitosan also affected the enzymatic activity of bitter gourd by enhancing the salt stress potential under increasing salt stress.

## Data Availability

The original contributions presented in the study are included in the article/supplementary material. Further inquiries can be directed to the corresponding authors.
